# Place identity among Vietnamese women in Taiwan: educational challenges and opportunities for integration

**DOI:** 10.3389/fpsyg.2025.1703995

**Published:** 2026-01-15

**Authors:** Chia-Fen Lin, Yi-Huang Shih

**Affiliations:** 1 National Taipei University of Education, Taipei City, Taiwan; 2 Minghsin University of Science and Technology, Xinfeng/Hsinchu, Taiwan

**Keywords:** culture, education, integration, place identity, transnational marriage, Vietnamese immigrant women

## 1 Introduction

Since the lifting of martial law in 1987, the rise of transnational marriages has led to the settlement of immigrant women from Mainland China, Vietnam, Indonesia, Hong Kong, Macao, and the Philippines in Taiwan, forming an emerging and diverse social group. These women have brought with them rich and heterogeneous cultural backgrounds that not only reshape family dynamics but also engender negotiations and tensions in child-rearing practices. At a broader societal level, the incorporation of diverse cultural traditions has contributed to the transformation of Taiwan's multicultural landscape. The arrival of immigrant women has introduced new lifestyles, languages, and cultural values, positioning Taiwan, within the context of globalization, as an increasingly inclusive and pluralistic society. Despite these contributions, immigrant women face considerable challenges in the process of adaptation, including language barriers, cultural differences, and social integration. In response, both the government and civil society have gradually recognized the significance of these challenges and implemented a range of supportive measures, such as language-learning programs, cultural adaptation initiatives, and campaigns to enhance societal understanding and inclusivity. With the growing number of transnational families, immigrant cultures have also begun to influence the education and upbringing of the next generation in Taiwan. This dynamic compels schools and communities to reconsider how to promote education and social integration in multicultural contexts, thereby developing a more inclusive social atmosphere and further enriching Taiwan's cultural and social fabric (Chuo, 2019; National Immigration Agency, 2023; Shih, 2020; Wang, 2001).

Vietnam has become one of the major source countries of women entering Taiwan through transnational marriage. Whether arriving through marriage, employment, or other migration pathways, these women encounter multiple challenges shaped by language barriers, cultural differences, social structures, and gender roles. The concept of place identity refers to the sense of belonging that individuals construct in relation to their environment within specific spatial and social contexts. For immigrant women, the formation of place identity is not only constrained by local social networks but also influenced by transnational connections and gender expectations. Nevertheless, existing studies have predominantly focused on “immigrant mothers” and their roles in their children's education, while relatively little attention has been given to the broader processes through which female immigrants construct place identity. (Yu 2016) conducted a study on the expectation gap and identity of Vietnamese female immigrants in Taiwan. The findings indicate that participants who failed to develop an “emotional attachment” to Taiwan were also the only individuals unable to construct a sense of identification with the host society. This suggests that emotional belonging constitutes a decisive determinant in the formation of identity, exerting a stronger influence than economic conditions, living environments, or political factors. (Tesng 2023) explored the process through which Vietnamese immigrant women in southern Taiwan construct their self-identities. Tesng's study also makes a significant contribution to the dialogue between place identity and self-construction theory. Tesng conceptualizes the formation of self-identity as a form of localized practice, emphasizing the interconnections among geographical space, social structures, and emotional bonds. This perspective helps illuminate how immigrant women shape their sense of belonging and cultural identity through lived local experiences, offering profound insights into the formation of Taiwan's multicultural society.

In particular, when examining the place identity of Vietnamese immigrant women in Taiwan, it is crucial to situate the analysis within the everyday contexts of early childhood education—namely, the interplay of preschool, family, and community in shaping maternal practices, language choices, and emotional attachments. Place identity is not a static label but rather a dynamic process of negotiation and reconstruction through spatial practices (e.g., school drop-off and pick-up, campus interactions), relational networks (teachers, peer parents, community organizations), and institutional discourses (curricula, assessment, parental expectations). For Vietnamese mothers, this process is simultaneously embedded in intersecting power relations of gender, class, and ethnicity, which further influence their parenting approaches and self-positioning (Anthias, 2012; Berry, 1997; Gustafson, 2001; Lan, 2008; Le Anh Huynh and Quoc Huynh, 2020; Proshansky et al., 1983; Wang, 2004; Wang and Shih, 2022). Against this backdrop, the present article focuses on Vietnamese immigrant women residing in Taiwan, examining the challenges and accessible opportunities they encounter in raising young children.

## 2 Method

Concepts serve as abstract mental constructs, mental images of phenomena, units of meaning, or building blocks of theory, intended to summarize specific aspects or elements of the human experience. However, for theory to be grounded in, and arise from real-world nursing practice, it is essential to bring clarity to the concepts under examination, known as concept analysis. Within the field of education, concept analysis has been extensively applied in theoretical development, curriculum design, and pedagogical practice, with the aim of rendering abstract concepts more concrete and operationalizable, thus fostering both theoretical advancement and pedagogical innovation. This article seeks to undertake a semantic analysis of one pivotal educational concept: place identity, and aims to examine the multiple meanings this concept assumes in different discursive environments, emphasizing their fluidity, cultural specificity, and the potential implications for educational practice. Through this analytical inquiry, the article aspires to contribute to a more nuanced understanding of the interconnections among place identity and educational challenges and opportunities in educational contexts for Vietnamese immigrant women in Taiwan (Gunawan et al., 2023; Morse, 2016; Penrod and Hupcey, 2005; Smith and Mörelius, 2021).

## 3 Place identity

(Proshansky et al. 1983) conceptualize place identity as a component of self-identity that emerges through individuals' ongoing interactions with specific environments, encompassing emotional attachment, processes of meaning construction, and a sense of belonging. Building on this foundation, (Massey 1994) and (Cresswell 2014) contend that place identity should be understood as inherently dynamic, relational, and infused with power relations, rather than as a stable or fixed attribute.

Place identity, develops based on the transactional—on people's side: perceptual and cognitive—processes between people and their environment and describes the phenomenon when places important to a person become related to their self-identity (Berze and Dúll, 2024). Place identity is a versatile concept upon which many psychological theories of human–environment relations are developed. The social constructivist theory of place identity sheds light on individuals' subjective perceptions of geographical space, providing valuable insights for studies of various disciplines, such as geography, sociology, psychology, environmental sciences and ecology, public administration, spatial planning, and so on. Place identity typically refers to the meaning and significance individuals attach to particular places, often including emotional connections, memories, and the sense of belonging to a specific location. It's about how people perceive and experience the physical environment, including cities, neighborhoods, or even specific buildings (Peng et al., 2020; Shih, 2024). Empirical studies indicate that a strong sense of place identity can both challenge and enrich educational adaptation. On the one hand, cultural dissonance, language barriers, and differing pedagogical beliefs may create tensions in the school–family interface. On the other hand, these same intercultural experiences can foster opportunities for multicultural understanding, broaden curricular perspectives, and enhance children's intercultural competence (Akkerman and Meijer, 2011; Berry, 1997; Kwon, 2017). Thus, examining immigrant mothers' place identity provides a valuable lens for understanding the intersection of culture, identity, and education in increasingly diverse societies.

## 4 How place identity specifically influences, shapes, or interacts with educational challenges and opportunities for Vietnamese immigrant women in Taiwan

Place identity is a versatile concept upon which many psychological theories of human–environment relations are built. Place identity refers to an individual's affective attachment, sense of belonging, and identification with a particular geographical or sociocultural space. For immigrant mothers, however, place identity extends beyond geographical affiliation; it encompasses the dynamic reconstruction of cultural memory, linguistic practice, social networks, and self-concept. Within intercultural contexts, an individual's sense of place identity profoundly shapes multiple dimensions of educational engagement. Specifically, it influences: (1) how immigrant mothers conceptualize the purposes and values of early childhood education; (2) the extent to which they adapt to the prevailing school culture and curricular expectations; (3) the ways in which they transmit and sustain their cultural heritage through family education; and (4) the quality of their communication and collaboration with teachers and other parents. Consequently, place identity may give rise to challenges in educational adaptation—such as cultural dissonance, linguistic barriers, and differing pedagogical beliefs—while simultaneously generating opportunities for promoting multicultural understanding, enriching curricular experiences, and fostering children's intercultural competence (Peng et al., 2020; Proshansky, 1978).

## 5 Discussion

### 5.1 Educational challenges

#### 5.1.1 Language barriers

Language proficiency has long been regarded as one of the core conditions for immigrant adaptation. Vietnamese immigrant women in Taiwan often face significant challenges due to limited competence in Mandarin Chinese, particularly in reading and writing. In addition to Mandarin, Taiwan's linguistic diversity—including Taiwanese Hokkien, Hakka, and Indigenous languages—further complicates their daily interactions, employment opportunities, and child-rearing practices. Even when they are able to communicate in Mandarin, Vietnamese-accented speech often becomes a salient marker of difference and a trigger for discrimination, subjecting them to linguistic prejudice in workplaces and social contexts. This phenomenon highlights that language is not merely a neutral communicative tool but also a symbol of social identity and power relations (Berry, 1997; Bourdieu, 1991; Norton, 2013). The implications of these linguistic barriers directly relate to the construction of place identity and sense of belonging. Place identity refers to the meanings, attachments, and self-conceptions individuals develop within particular sociocultural and spatial contexts. When immigrant mothers are stigmatized because of limited Mandarin proficiency or accented speech, their opportunities to build meaningful connections with local communities are diminished, thereby weakening their sense of belonging in Taiwanese society. Furthermore, linguistic exclusion reinforces their outsider status, signaling that they are not fully integrated into the dominant cultural and linguistic order (Antonsich, 2010; Proshansky et al., 1983). Thus, language proficiency—or its absence—functions not only as a practical determinant of immigrant adaptation but also as a symbolic boundary that shapes whether immigrant women can negotiate identity, foster belonging, and establish rootedness within Taiwanese society.

#### 5.1.2 Cultural differences, discrimination, and place identity

Culture has a variety of definitions; it has been defined as the spiritual creation of human beings, the material civilization created by human beings, or some combination of the two. Culture refers to a group of people's way of life, including their religion, food, clothing, language, rites of passage, and music. Cultural differences exist within society. The adaptation processes of immigrant mothers in Taiwan through cross-national marriage are often shaped by cultural differences and experiences of discrimination. In everyday practices such as gender roles, child-rearing, and dietary habits, immigrant women frequently encounter social stereotypes and cultural exclusion. These stereotypes not only construct Southeast Asian women as “submissive” or of “low socioeconomic status,” but also constrain their agency within both family and societal interactions. Moreover, discriminatory experiences extend beyond the individual to affect their children. When children face exclusion in schools or peer groups due to their “mixed-race” identities or their mothers' immigrant backgrounds, mothers bear the burden of what has been described as vicarious discrimination. Such experiences are closely tied to questions of place identity and sense of belonging. Place identity refers to the meanings and emotional attachments individuals develop in relation to particular social and spatial contexts. For immigrant mothers, the persistence of cultural stereotypes and discriminatory encounters often disrupts their ability to form a secure attachment to their host society, thereby weakening their sense of belonging. The tension between cultural difference and social exclusion illustrates that immigrant mothers' adaptation is not solely an individual matter of adjustment but is deeply embedded within broader structures of power, cultural recognition, and identity negotiation (Anthias, 2012; Antonsich, 2010; Cockel, 2013; Lan, 2008; Shih, 2022; Proshansky et al., 1983; Wang, 2004).

Consequently, cultural differences and discrimination should be understood not only as sources of personal or familial stress but also as significant factors influencing immigrants' capacity to construct place identity and achieve a meaningful sense of belonging in Taiwanese society. This insight highlights that the experiences of Vietnamese immigrant women in Taiwan cannot be fully understood through psychological adjustment alone; rather, they must be situated within broader sociocultural and structural contexts. Cultural differences often intersect with systemic inequalities—such as linguistic hierarchies, gender norms, and power relations—that shape immigrants' opportunities to participate in community life and to be recognized as legitimate members of society. Discrimination, whether overt or subtle, can hinder immigrants' efforts to establish emotional and symbolic connections to their living environment, thereby obstructing the process of place identity formation. Conversely, when inclusive social spaces and intercultural dialogue are fostered, immigrants can transform these challenges into opportunities for mutual understanding and transcultural identity development. In this sense, Tesng's (2023) findings underscore that belonging is not merely a matter of personal adaptation but a relational and political process—one that depends on both the agency of immigrant women and the openness of Taiwanese society to cultural diversity. Thus, promoting intercultural respect, equitable participation, and recognition of multiple cultural identities becomes central to enhancing immigrants' wellbeing and social integration.

### 5.2 Educational opportunities: multicultural education policies create a discursive space that enables individuals to renegotiate and reconstruct their place identities in the future

Multicultural education policies, while providing discursive spaces for individuals to renegotiate and reconstruct their identities, are not ideologically neutral. Such policies are frequently embedded within dominant national narratives and political frameworks, which both enable and constrain transformative possibilities. From a critical pedagogy perspective, multicultural education holds the potential to challenge hegemonic cultural discourses and legitimize marginalized voices, fostering social justice and empowerment (Banks, 2016; Freire, 1970; Giroux, 1992). However, as (Sleeter 2024) notes, multicultural education often risks being reduced to symbolic gestures that emphasize cultural celebrations or superficial diversity while leaving structural inequities unaddressed. Empirical studies in the Taiwanese context illustrate this dilemma. For instance, research on new immigrant education has shown that policies frequently highlight cultural representation—such as food festivals or heritage days—yet provide insufficient attention to more pressing issues, including immigrant mothers' linguistic rights, children's integration into mainstream schooling, and unequal access to social resources. Consequently, while such policies may increase visibility and recognition for new immigrant communities, they may simultaneously reproduce existing hierarchies by confining diversity to symbolic domains rather than addressing systemic inequalities. This tension raises a critical question: does multicultural education in Taiwan function as a pathway toward emancipation, or does it serve as a mechanism of domestication that regulates diversity within socially acceptable boundaries? Answering this question requires integrating both theoretical insights from critical pedagogy and empirical investigations of local educational practices in the future in Taiwan (Banks, 2016; Shih, 2020; Shih and Wang, 2022; Sleeter, 2024).

## 6 Reflections: rethinking the close and dynamic conceptual linkages among place identity, educational challenges, and processes of integration.

There are close and dynamic conceptual linkages among place identity, educational challenges, and processes of integration. Place identity—defined as an individual's emotional attachment, meaning-making, and sense of belonging to a particular geographical setting (Proshansky et al., 1983)—significantly shapes how individuals participate, position themselves, and negotiate identities within educational contexts. For immigrant women, when place identity is still emerging or unstable, they often face greater uncertainty in language practices, cultural understanding, and social interaction. These forms of uncertainty give rise to concrete educational challenges, including linguistic barriers, cultural incongruence, and limited familiarity with institutional norms (Berry, 1997; Suárez-Orozco et al., 2010). Such educational challenges directly influence immigrant women's processes of integration into both everyday life and school systems.

Integration has become both a key policy objective related to the resettlement of refugees and other migrants, and a matter of significant public discussion. Integration is not merely a matter of adapting to new cultural expectations but represents a bidirectional, relational process shaped by the resources, recognition, and opportunities provided by receiving institutions. When educational institutions offer culturally responsive, linguistically accessible, and supportive learning environments, immigrant women are better positioned to reconstruct their place identity through learning, social interaction, and participation in community life. This strengthens their sense of belonging to Taiwan. Conversely, when schools fail to address cultural differences, linguistic needs, or structural exclusions, immigrant women's experiences of marginalization intensify, hindering their integration trajectories (Ager and Strang, 2008; Bankston and Zhou, 2002). Thus, place identity, educational challenges, and integration processes form a mutually constitutive cycle: place identity refers to a person's sense of belonging to a particular culture, place or group, and shapes educational experiences, educational experiences reshape place identity, and together they determine the possibilities and directions of immigrant integration. This conceptual linkage underscores the importance of considering emotional belonging, cultural interaction, and institutional structures simultaneously when examining the educational experiences of immigrant women. Attending to these interconnected dimensions enables a fuller understanding of the complexities, negotiations, and structural conditions that shape immigrant integration (Proshansky et al., 1983; Shih, 2022).
